# Brain Aging and AD-Like Pathology in Streptozotocin-Induced Diabetic Rats

**DOI:** 10.1155/2014/796840

**Published:** 2014-08-14

**Authors:** Jian-Qin Wang, Jie Yin, Yan-Feng Song, Lang Zhang, Ying-Xiang Ren, De-Gui Wang, Li-Ping Gao, Yu-Hong Jing

**Affiliations:** ^1^Nephrology Department and Blood Dialysis Center, Second Hospital of Lanzhou University, Lanzhou 730000, China; ^2^Institute of Anatomy and Embryology, School of Basic Medical Sciences, Lanzhou University, Lanzhou 730000, China; ^3^Key Laboratory of Preclinical Study for New Drugs of Gansu Province, Lanzhou University, Lanzhou 730000, China; ^4^Institute of Biochemistry and Molecular Biology, School of Basic Medical Sciences, Lanzhou University, Lanzhou 730000, China

## Abstract

*Objective.* Numerous epidemiological studies have linked diabetes mellitus (DM) with an increased risk of developing Alzheimer's disease (AD). However, whether or not diabetic encephalopathy shows AD-like pathology remains unclear. *Research Design and Methods.* Forebrain and hippocampal volumes were measured using stereology in serial coronal sections of the brain in streptozotocin- (STZ-) induced rats. Neurodegeneration in the frontal cortex, hypothalamus, and hippocampus was evaluated using Fluoro-Jade C (FJC). A*β* aggregation in the frontal cortex and hippocampus was tested using immunohistochemistry and ELISA. Dendritic spine density in the frontal cortex and hippocampus was measured using Golgi staining, and western blot was conducted to detect the levels of synaptophysin. Cognitive ability was evaluated through the Morris water maze and inhibitory avoidant box. *Results.* Rats are characterized by insulin deficiency accompanied with polydipsia, polyphagia, polyuria, and weight loss after STZ injection. The number of FJC-positive cells significantly increased in discrete brain regions of the diabetic rats compared with the age-matched control rats. Hippocampal atrophy, A*β* aggregation, and synapse loss were observed in the diabetic rats compared with the control rats. The learning and memory of the diabetic rats decreased compared with those of the age-matched control rats. *Conclusions.* Our results suggested that aberrant metabolism induced brain aging as characterized by AD-like pathologies.

## 1. Introduction

Global incidence of diabetes mellitus (DM) estimates more than 171 million for 2000 and 366 million for 2030 [1]. The mortality and morbidity of DM are determined by various complications, such as diabetic vasculopathy, retinopathy, nephropathy, and peripheral neuropathy [2]. Recently, many studies have indicated that DM also implicated the central nervous system (CNS) and induced the brain pathological changes, named the diabetic encephalopathy, which is a complication of DM in the CNS characterized by mild cognitive deficits and neuropathology [3–5]. Diabetic encephalopathy presents many symptoms, which can be described as the features of brain aging including brain atrophy, reactive oxygen species (ROS) accumulation, cerebral vasculopathy, and impairment of cognition [6, 7]. Clinical observation has shown that brain atrophy is more remarkable in diabetic patients than in age-matched controls [8]. Animal experimental data have suggested that learning deficiency is associated with the distinct changes in synaptic plasticity in hippocampal slices in streptozotocin- (STZ-) induced diabetic rats [9]. The affinity of glutamate for AMPA but not for NMDA receptors decreases in Sprague-Dawley (SD) rats at 6 to 8 weeks after STZ injection [10]. The levels of malondialdehyde, xanthine oxidase, and nitric oxide in the hippocampus, cortex, cerebellum, brain stem, and spinal cord significantly increase in STZ-induced diabetic-untreated rats, suggesting remarkable generation of ROS in the brain [11].

Hyperglycemia resulting from defective insulin secretion, resistance to insulin action, or both is a critical pathogenesis of DM. Hyperglycemia is linked to all chronic DM complications. Willem Hendrik Gispen and Geert-Jan Biessels have recently suggested that acute hyperglycemia is associated with mild cognitive dysfunction in population with type 1 or type 2 DM [12]. Another recent study has suggested that insulin is implicated in the pathogenesis of age-related memory decline and diabetic encephalopathy [13, 14]. Insulin may act as a neuromodulator that regulates the release and reuptake of neurotransmitters and probably affects learning and memory [15]. Impairments in the insulin signaling pathway in the periphery and brain have been implicated in Alzheimer's disease, diabetes, and aging [16, 17].

Recent studies have revealed that impairments in cerebral glucose utilization and energy metabolism represent early abnormalities that precede or accompany the initial stages of cognitive impairment [18]. Cerebral glucose utilization deficiency and insulin signaling decline are common features between DM and Alzheimer's disease (AD) [19]. AD is a progressive neurodegenerative disease characterized by the loss of memory and other cognitive functions, resulting in dementia. The hallmarks of pathology of AD are A*β* deposition and microtubule-associated protein tau overphosphorylation and formed the senile plaques in the extracellular matrix [20]. Additionally, some studies have indicated that the insulin signals are involved in regulation of A*β* accumulation and tau phosphorylation [17, 21]. And epidemiological surveys suggested that diabetes is associated with an increased prevalence of AD [22]. Furthermore, the factors associated with high risk of AD are also involved in the development of DM, especially T2DM [23]. Therefore, some literatures have proposed that AD represents “type 3 diabetes” [24].

Clinical and experimental data clearly showed that diabetes affected the brain and these effects are similar to the acceleration of brain ageing. However, whether or not diabetic encephalopathy at early onset shows AD-like pathology as aging dependent neurodegenerative disease remains unclear. In the present study, SD rats were inflicted with hyperglycemia and insulin deficiency through STZ injection. Metabolic parameters were observed consecutively, and anatomic changes in the brain were analyzed. Changes in the brain were indicated by hippocampal atrophy accompanied with beta-amyloid deposition, synapse loss, and learning behavioral deficiency at 4 months after STZ injection.

## 2. Materials and Methods

### 2.1. Animals and Reagents

Fiftymale Sprague-Dawley (SD) rats (8–10 weeks old) were obtained from the Experimental Animal Center of Lanzhou University. The rats were kept in an animal house at 22 ± 2°C temperature, 65 ± 10% relative humidity, and 12 h light/dark cycle. The animals were provided with food and water* ad libitum*. All experimental protocols were approved by the Institutional Animal Ethic Committee, Lanzhou University. Efforts were exerted to minimize animal suffering and reduce the numbers of animals used. STZ was purchased from Sigma (St. Louis. MO, USA). Fluoro-Jade C (FJC) and mouse monoclonal anti-GAPDH and rabbit polyclonal anti-A*β*42 antibodies were purchased from Millipore (Bellerica, MA, USA). Glucose, total cholesterol, triglyceride, and creatine enzymatic diagnostic kits were purchased from Randox (Crumlin, County Antrim, UK). Rabbit polyclonal anti-synaptophysin (SYN) antibody was purchased from Santa Cruz Biotechnology Incorporation (Santa Cruz, CA, USA). A*β*42 ELISA kits were purchased from R&D systems, Inc. (Emeryville, CA, USA).

### 2.2. Experimental Procedure

Thirty rats were fasted overnight and then injected with STZ (65 mg/kg body wt.) through the femoral vein. Twenty age-matched normal rats received equivalent volume of normal saline. One week after STZ injection, blood samples were collected through the tail vein, and plasma glucose level and insulin level were measured by Plasma Glucose Enzymatic Diagnostic Kits and insulin ELISA Kit, respectively. Rats with the plasma glucose level ≥300 mg/dL and symptoms of polyuria, polyphagia, and polydipsia were considered to be diabetic and used in the present study. The fasting plasma insulin level in STZ-induced DM rats was 1.32 ± 0.6 pmol/L and was markedly lower than that of the normal rats (166.2 ± 4.3 pmol/L).

### 2.3. Metabolic Analysis

The metabolic parameters were tested according to our previous methods [25]. In brief, the rats were placed in metabolic cages and raised for 24 h after 8 h of fasting. Urine/24 h, water/24 h, and food consumption/24 h were measured, and fasting plasma glucose, triglyceride, total cholesterol, and creatinine were quantified using respective enzymatic diagnostic kits. Creatinine clear ratio (CCR) was calculated according to urine volume/24 h and body weight; the CCR was calculated as follows:

(1) $$\begin{array}{c} \frac{\text{Urine}\;\text{creatinine}\times \text{urine}\;\text{volume}\;\left(\text{mL}/24\;\text{h}\right)}{\left(\text{Plasma}\;\text{creatinine}\times 1440\right)} \end{array}$$

and then normalized by body weight.

Metabolic analysis was performed one time a month and detected for four consecutive months. Incomplete data was deleted during metabolic analysis. Finally, the total metabolic data of 18 rats in each group were recorded after four months.

### 2.4. Tissue Preparation

Six rats in each group were sacrificed with an overdose of 10% chloral hydrate and then transcardially perfused with 0.9% saline solution followed by 4% ice-cold phosphate-buffered paraformaldehyde (PFA). The brains were removed, postfixed in 4% PFA for 12 h, and then immersed sequentially in 20% and 30% sucrose solutions in 0.1 M phosphate buffer (pH 7.4) until they sank. Coronal sections with a thickness of 40 *μ*m were cut at 2.2 mm to −4.80 mm from the bregma using a freezing microtome (Leica, Germany) and then stored at −20°C in a cryoprotectant solution.

### 2.5. Measurement of Forebrain and Hippocampal Volumes

To evaluate brain atrophy, the forebrain and hippocampal volumes were measured according to our previous methods [26]. In brief, for forebrain volume, six sections at 2.28 mm to −0.12 mm from the bregma (interval at 400 *μ*m) were selected and then stained with cresyl violet. The sections were observed and photographed under a stereoscopic microscope. Frontal cortex areas were measured using Image J software. The forebrain volume was calculated as

(2) [S1a+S2a2+S2a+S3a2+S3a+S4a2 +S4a+S5a2+S5a+S6a2]×400,

where S1a to S6a represent the forebrain areas in sections 1 to 6, respectively.

For hippocampal volume, six sections at −2.2 mm to −4.6 mm from the bregma (interval at 400 *μ*m) were selected and then stained with cresyl violet. The sections were observed and photographed under a stereoscopic microscope. Hippocampal areas were measured using Image J software. The hippocampal volume was calculated as

(3) [S1a+S2a2+S2a+S3a2+S3a+S4a2 +S4a+S5a2+S5a+S6a2]×400,

where S1a to S6a represent the hippocampal areas in sections 1 to 6, respectively.

### 2.6. FJC Staining

To evaluate the neurodegeneration, the FJC staining was used according to our previous methods [26]. In brief, six sections at 2.28 mm to −0.12 mm and six sections at −2.2 mm to −4.6 mm from the bregma (at 400 *μ*m intervals) were selected, respectively. FJC staining and imaging analysis were performed as previously described. Dried sections were dipped in 80% ethanol solution that contains 1% sodium hydroxide, 70% ethanol, and 0.06% potassium permanganate for 5, 2, and 10 min, respectively. The sections were rinsed with distilled water and then incubated with 0.0004% FJC in 0.1% acetic acid for 20 min. FJC staining was detected under a fluorescent microscope at 480 nm excitation and 525 nm emission. Images were acquired through a 20× objective, and the number of FJC-positive cells in the frontal cortex, hippocampus, and hypothalamus was counted.

The total number of positive cells in the frontal cortex was calculated as

(4) $$\begin{array}{c} \left[\frac{\text{S}1+\text{S}2}{2}\;+\frac{\text{S}2+\text{S}3}{2}+\frac{\text{S}3+\text{S}4}{2}+\frac{\text{S}4+\text{S}5}{2}+\frac{\text{S}5+\text{S}6}{2}\right]\times 10, \end{array}$$

where S1 to S6 represent the FJC-positive cell numbers in sections 1 to 6, respectively.

The total number of positive cells in the hippocampus and hypothalamus was calculated as

(5) [S7+S82+S8+S92+S9+S102 +S10+S112+S11+S122]×10,

where S7 to S12 represent the FJC-positive cell numbers in hippocampus or hypothalamus in sections 7 to 12, respectively.

### 2.7. Immunohistochemistry

For A*β*42 immunostaining, three sections at 2.28 mm, −0.12 mm, and −2.2 mm from the bregma were selected and then incubated with 0.3% H_2_O_2_ for 30 min. The sections were placed in blocking buffer that contains 10% normal goat serum and 0.3% triton X-100 in 0.01 M phosphate-buffered saline (pH 7.2) for 30 min at 37°C and then incubated overnight with antibodies against rabbit polyclonal anti-A*β*42 (1 : 500) at 4°C. The sections were then incubated with corresponding biotinylated secondary antibodies (1 : 200) at 37°C for 1 h and then with avidin-biotin-peroxidase (1 : 200) at 37°C for 1 h. Immunoreactivity was visualized with 0.05% 3, 3′-diaminobenzidine as chromogen. Negative controls received the same treatment without the primary antibodies and showed no specific staining.

### 2.8. A*β*42 ELISA

The content of A*β*42 in hippocampus was tested according to the previous methods [27]. Six rats were sacrificed by decapitation, and their brains were quickly removed and placed on ice-cold glass plates. The hippocampus was rapidly dissected, frozen, and then stored in a deep freezer at −80°C until assayed. The frozen tissues were homogenized in 4 volumes of buffer A that contains 50 mM Tris-HCl (pH 7.6), 150 mM NaCl, and a protease inhibitor cocktail (Complete; Roche Diagnostics, Mannheim, Germany) with 10 strokes of a Teflon glass homogenizer. The tissues were centrifuged at 20,000 g for 20 min at 4°C. The supernatant was used as the soluble fraction, and the pellet was solubilized by sonication in buffer A that contains 6 M guanidine-HCl. The solubilized pellet was centrifuged at 20,000 g for 20 min at 4°C. The supernatant was diluted 12-fold to reduce the concentration of guanidine-HCl and used as the insoluble fraction. The amount of A*β*42 in each fraction was determined by sandwich ELISA.

### 2.9. Golgi Staining and Dendritic Spine Analysis

Golgi staining was performed on 4-month-old diabetic rats and age-matched normal rats according to the previous methods [28]. Four rats were randomly selected from each group. Briefly, freshly dissected brains were immersed in a Golgi-Cox solution for 2 weeks at room temperature. The Golgi-Cox solution was replaced, and immersion was continued for 2 weeks. The pyramidal neurons in cortical layers II/III and in the CA1 region of the hippocampus were analyzed. Five neurons were randomly selected per region in each rat. A minimum of two segments were randomly selected per neuron from the apical oblique and basal shaft dendrites. Dendritic spine density was measured in a blinded manner.

### 2.10. Measurement of RNA and Protein Concentrations

Fourrats in each group were sacrificed by decapitation, and their brains were quickly removed and placed on ice-cold glass plates. The cerebral cortex was dissected and frozen in liquid nitrogen. Total RNA in the left cerebral cortex was extracted by Trizol reagent, and RNA concentration was measured using a spectrofluorometer. Total protein in the right cerebral cortex was extracted by RIPA buffer, and protein concentration was measured using the Bradford assay.

### 2.11. Western Blot Analysis

Four rats were randomly selected from each group and then anesthetized with pentobarbital. The frontal cortex and hippocampus of the rats were placed on ice-cold glass plates. The samples were frozen in liquid nitrogen and then homogenized. Total protein was extracted using RIPA buffer that contains protease inhibitors. Proteins (50 *μ*g) were fractionated on 10% sodium dodecyl sulfate-polyacrylamide gel electrophoresis and then transferred into polyvinylidene fluoride membranes. The membranes were blotted with anti-synaptophysin (1 : 1000) and anti-GAPDH (1 : 5000) antibodies, as well as with horseradish peroxidase-conjugated second antibody (1 : 5000). Immunoreactive protein bands were visualized by enhanced chemiluminescence.

### 2.12. Morris Water Maze

Morris water maze was performed as previously described [29]. Briefly, at 4 months after STZ injection, twelve rats were selected according to the open field test and then allowed to perform a learning task in the Morris water maze with the blind method. The maze consisted of a black pool (148 cm diameter) filled with water (26 ± 2°C). A circular black platform was submerged 2 cm below the water surface in the middle of the target quadrant. The behavior of the rats in the pool was traced with a camera connected to a WMT-100 analysis system (Taimeng, Chinese Instruments). The swimming speed, distance, and latency of the rats to find the platform were measured. The pool was divided into four quadrants (i.e., quadrants 1 to 4). The quadrants had different shapes, different colors, and three rings (inner, middle, and outer). The platform was placed in a constant location in the middle ring of quadrant 3. The rats were trained for 4 d with the hidden platform. Each day involved training the rats in the four quadrants with 30 min intervals. Each trial was started by placing a rat with its back facing toward the platform at the starting points. The trial was terminated when the rat stood on the platform. However, when the rat did not find the platform within 60 s, it was guided on the platform for 15 s. During acquisition (days 1 to 4) of the spatial navigation test, all groups were given one session of four trials each day. On each day after training, the rat was removed from the pool, dried, and then returned to its cage.

### 2.13. Inhibitory Avoidance

IA was performed as previously described [30]. Briefly, the IA box consisted of a lighted (safe) compartment and a dark (shock) compartment separated by a door. In the dark compartment, the rats received a footshock of 0.6 mA for 2 s through a constant current scrambler circuit delivered through the grid floor. Rats were placed in the lighted compartment, as specified in each experiment, facing away from the door. The door leading to the dark chamber was opened after 10 s. Once the animals fully entered the dark chamber (all four limbs contacting the grid in the dark chamber), the door was closed, and a footshock was delivered for 2 s after a 2 s delay. The rats were returned to their home cage 10 s later and were tested 48 h after training. The latency of the rats to enter the dark compartment was recorded.

### 2.14. Statistical Analysis

All data were expressed as mean ± SEM. Differences between groups were analyzed by ANOVA. Mann-Whitney* U* test was used for each evaluation, and unpaired Student's* t*-test was used for other data. Statistical significance was considered at *P* < 0.05.

## 3. Results

### 3.1. Metabolic Parameters

**Figure 1:**
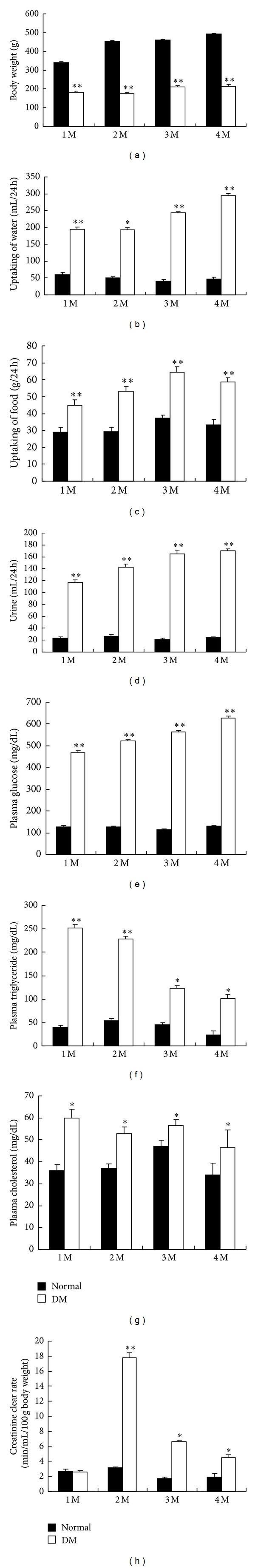
Metabolic parameters of rats at four consecutive months after STZ injection. (a) Body weight, (b) water absorption/24 h, (c) food absorption/24 h, (d) urine/24 h, (e) plasma glucose, (f) plasma triglyceride, (g) plasma cholesterol, (h) creatinine clear ratio. *P < 0.05 denotes significant difference compared with the age-matched control rats. **P < 0.01, significant difference compared with the age-matched control rats; *n* = 18.

The results of consecutive metabolic test showed that the body weight (Figure 1(a)) of the diabetic rats was reduced compared with that of the age-matched rats. This test also demonstrated that the water consumption (Figure 1(b)), food consumption (Figure 1(c)), and urine production (Figure 1(d)) of the diabetic rats increased compared with those of the age-matched rats. Consecutive plasma examination showed that the levels of glucose (Figure 1(e)), triglyceride (Figure 1(f)), and cholesterol (Figure 1(g)) were higher in the diabetic rats than in the age-matched rats. Moreover, CCR was higher in the diabetic rats than in the age-matched control rats; this result was more pronounced at 2 months after STZ injection, suggesting that the kidney function was normal and that muscle wasting increased in the diabetic rats (Figure 1(h)).

### 3.2. Changes in Brain Structure

**Figure 2:**
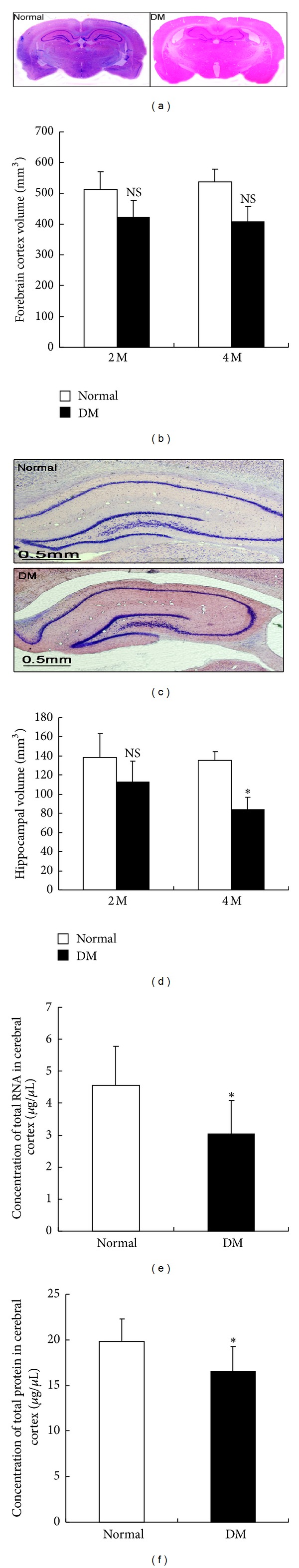
Changes in the brain sterostructure of STZ-induced diabetic rats. (a) Representative forebrain images at 4 months after STZ injection. (b) Forebrain volume measurement (see Materials and Methods for procedure details). (c) Representative images of rat hippocampus at 4 months after STZ injection. (d) Hippocampal volume measurement (see Materials and Methods for procedure details). *P < 0.05, significant difference compared with the age-matched control rats; *n* = 6. ((e) and (f)) Total RNA and protein concentrations were measured in whole brain at 4 months after STZ injection. *P < 0.05, significant difference compared with the age-matched control group; *n* = 4.

To evaluate the brain atrophy, forebrain and hippocampus volumes were measured. Results showedthe forebrain volume is not significantly different in the diabetic rats compared to the age-matched control rats at 2 and 4 months after STZ injection (Figures 2(a) and 2(b)). The hippocampal volume in the diabetic rats decreased at 4 months after STZ injection compared with that in the age-matched control rats (Figures 2(c) and 2(d)).

### 3.3. Changes in Total RNA and Protein Concentrations in Cerebral Cortex

The concentrations of RNA and protein are shown in Figures 2(e) and 2(f). The total RNA concentrationin the cerebral cortex of the diabetic rats decreased at 4 months after STZ injection (3.032 ± 1.05 *μ*g/*μ*L) compared with that in the cerebral cortex of the age-matched control rats (4.56 ± 1.21 *μ*g/*μ*L). The total protein concentration in the cerebral cortex of the diabetic rats also decreased at 4 months after STZ injection (16.535 ± 2.76 *μ*g/*μ*L) compared with that in the cerebral cortex of the age-matched control rats (19.805 ± 2.49 *μ*g/*μ*L).

### 3.4. Neurodegeneration in the Frontal Cortex, Hippocampus, and Hypothalamus

**Figure 3:**
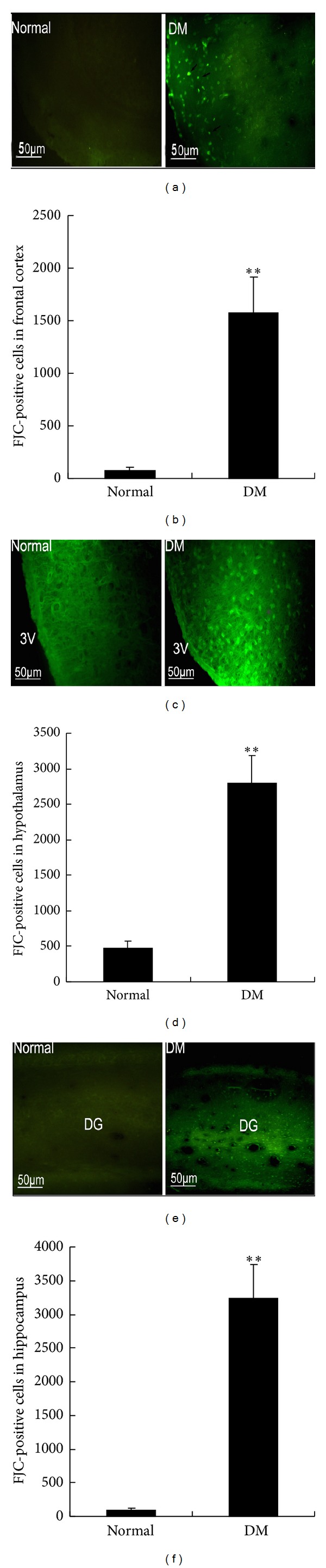
Fluoro-Jade C- (FJC-) positive cells were examined in the frontal cortex, hypothalamus, and hippocampus of rats at 4 months after STZ injection. (a) Representative images of FJC-positive cells in the frontal cortex. (c) Representative images of FJC-positive cells in the hypothalamus; 3V: third ventricle. (e) Representative images of FJC-positive cells in the hippocampus; DG: dentate gyrus. (b) Frontal cortex, (d) hypothalamus, and (f) hippocampus. FJC-positive cells were counted (see Materials and Methods for procedure details). **P < 0.01, significant difference compared with the age-matched control rats; *n* = 6.

The effect of the persistent metabolic disorder on neuronal survival was determined by conducting FJC staining to evaluate the neurodegeneration in discrete brain regions. The number of FJC-positive cells was higher in the frontal cortex (Figures 3(a) and 3(b), 1570 ± 350), hypothalamus (Figures 3(c) and 3(d), 2800 ± 385), and hippocampus (Figures 3(e) and 3(f), 3235 ± 502) of the diabetic rats at 4 months after STZ injection than in those of the age-matched control rats.

### 3.5. DM-Induced A*β*42 Deposition

**Figure 4:**
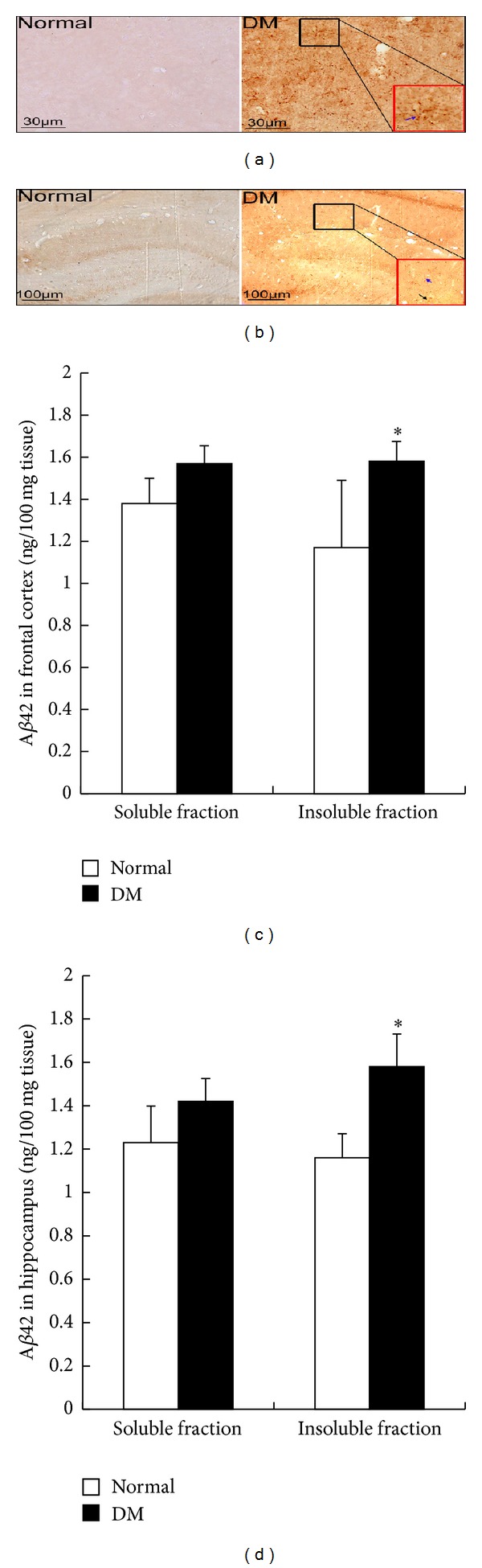
A*β*42 deposition in rat hippocampus at 4 months after STZ injection. (a) Representative images of A*β*42 immunoreaction in the frontal cortex: black square was magnified and shown in red square; blue arrow indicates the glial cells. (b) Representative images of A*β*42 immunoreaction in the hippocampus: black square was magnified and shown in red square, blue arrow indicates the glial cells, and black arrow indicates the neuron. (c) A*β*42 was quantified in soluble and insoluble fractions of the cortex by ELISA. (d) A*β*42 was quantified in soluble and insoluble fractions of the hippocampus by ELISA. *P < 0.05, significant difference compared with the age-matched control rats; *n* = 6.

A*β* deposition is the hallmark characteristic in AD and AD-like brain aging; in the present study,*A* *β* deposition was analysis. Immunohistochemistry revealed the presence of A*β*42 immunoreactivity in the frontal cortex (Figure 4(a)) and hippocampus (Figure 4(b)) of the diabetic rats at 4 months after STZ injection. Quantitative analysis of A*β*42 in the frontal cortex and hippocampal tissues by ELISA showed no significant difference in the soluble fraction of hippocampal tissues between the diabetic rats and age-matched control rats. However, the amount of A*β*42 in the insoluble fraction increased in the diabetic rats at 4 months after STZ injection compared with that in the age-matched control rats (Figures 4(c) and 4(d)).

### 3.6. Reduction of Dendritic Spine Density in Diabetic Rat

**Figure 5:**
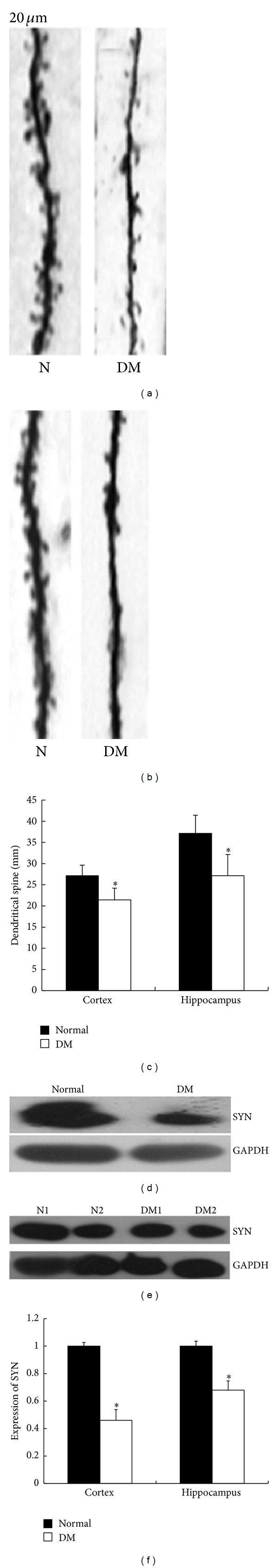
Decline in dendritic spine density in the frontal cortex and hippocampus of rats at 4 months after STZ injection. (a) Representative images of the dendritic spine of the pyramidal neurons in cortical layers II/III. (b) Representative images of the dendritic spine of the pyramidal neurons in the CA1 region of the hippocampus. (c) Dendritic spine density in the frontal cortex and hippocampus was measured (see Materials and Methods for procedure details). *P < 0.05, significant difference compared with the age-matched control rats; *n* = 4. (d) Representative images of synaptophysin expression in the frontal cortex using western blot analysis. (e) Representative images of synaptophysin expression in the hippocampus using western blot analysis. (f) Relative expression of synaptophysin in the frontal cortex and hippocampus was determined. *P < 0.05, significant difference compared with the age-matched control group; *n* = 4.

To evaluate the synaptic plasticity, dendritic spine density and SYN expression were measured. Results showed that the dendritic spine density of the frontal cortex decreased in the diabetic rats at 4 months after STZ injection compared with that in the age-matched control rats (Figures 5(a) and 5(c)). Consistently, the synaptophysin expression of the frontal cortex was lower in the diabetic rats than in the control rats (Figures 5(d) and 5(f)). Similarly, the dendritic spine density of the hippocampus decreased in the diabetic rats at 4 months after STZ injection compared with that in the age-matched rats (Figures 5(b) and 5(c)). The synaptophysin level in the hippocampal tissues decreased in the diabetic rats compared with that in the age-matched control rats (Figures 5(e) and 5(f)).

### 3.7. Learning and Memory Impairment in Diabetic Rats

**Figure 6:**
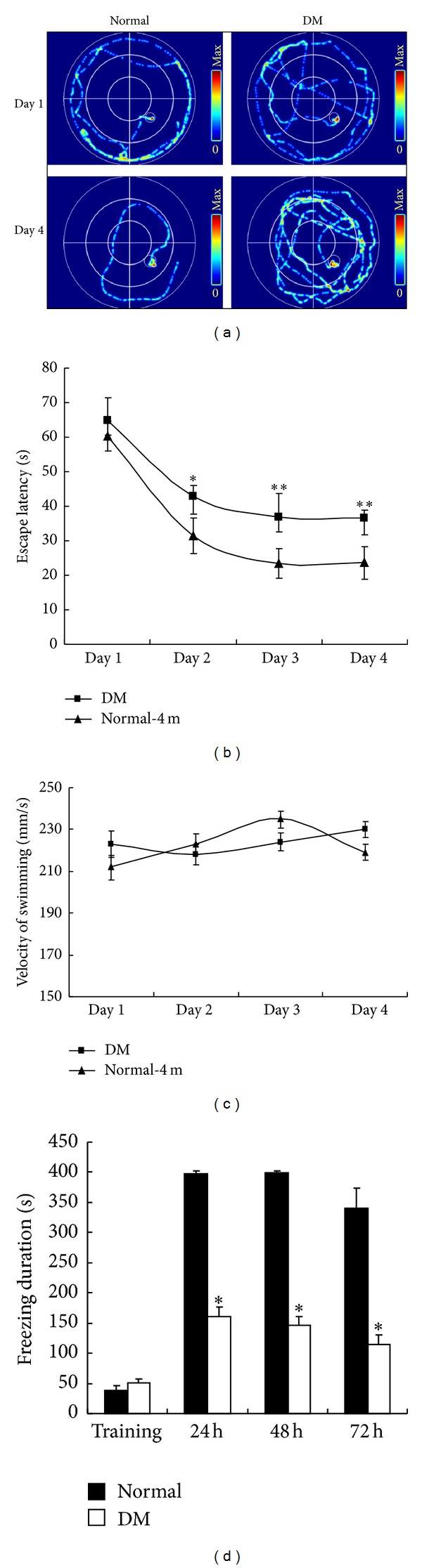
Evaluation of the cognition of diabetic rats at 4 months after STZ injection. (a) Representative traces by the Morris water maze. The performance of rats in the Morris water maze using hidden platform training for 4 d was assessed based on (b) escape latency and (c) swimming velocity in the normal and DM groups. (d) Freezing duration was measured using inhibitory avoidance box. *P < 0.05, significant difference compared with the age-matched control rats; **P < 0.01, significant difference compared with the age-matched control rats; *n* = 12.

At 1 d after training, no significant difference in the time to find the platform (escape latency) was found between the diabetic rats and age-matched control rats at 4 months after STZ injection. At 2, 3, and 4 d after training, the escape latency exhibited by the diabetic rats was significantly longer than that exhibited by the control rats (Figures 6(a) and 6(b)). The velocity of swimming has no significant difference in both groups (Figure 6(c)). No significant difference in training of IA was observed between the diabetic rats and age-matched control rats at 4 months after STZ injection. However, at 24, 48, and 72 h after training, the freezing duration exhibited by the diabetic rats was notably shorter than that exhibited by the control rats (Figure 6(d)).

## 4. Discussion

In this study, cognitive impairment accompanying hippocampal atrophy was observed at 4 months after STZ injection. Brain atrophy, as a general feature during brain aging, is involved in complicated mechanisms. STZ-induced animal models are characterized by insulin deficiency accompanied with polydipsia, polyphagia, polyuria, and weight loss. Insulin receptors are distributed in several brain regions, including the hippocampus, hypothalamus, olfactory bulb, and cortex. And plasma insulin can pass the blood-brain barrier [31], binding with its receptor to play important roles. In the CNS, insulin is involved not only in regulating the glucose metabolism, but also in maintaining the neuronal survival and development [32]. In the hippocampus, insulin signal is closely correlated with learning and memory behavior. The administration of insulin enhances performance in a passive-avoidance memory task, and spatial memory training alters the expression levels of insulin receptors in the hippocampus [33]. Consistently, recent animal studies have suggested that delivery of insulin to the hippocampus modulates hippocampal memory processes [34]. Therefore, insulin deficiency may play pilot roles in development of diabetic encephalopathy in STZ-induced animal model.

Our results indicated that the dendritic spine density in the frontal cortex and hippocampus decreased in the STZ-induced diabetic rats compared with the age-matched control rats. Synaptophysin (SYN), a crucial presynaptic protein, also decreased in the diabetic rats compared with age-matched control rats. Aside from hyperglycemia due to insulin deficiency, aberrant cholesterol metabolism also occurred in the STZ-induced rats [35]. Brain is the most cholesterol-rich organ in the body; most of this cholesterol is produced* in situ*. The notion that decreased cholesterol biosynthesis alters the brain function is supported by the results of* in vitro *studies that cholesterol is essential for synaptogenesis and synapse function. In our present experiment, the insulin deficiency and aberrant lipid metabolism may contribute to the decline of synaptic plasticity.

In the present study, A*β*42 deposition in the hippocampus was observed in astrocytes and neurons. However, it did not form plaques in the extracellular matrix at 4 months after STZ injection. At early onset, A*β* aggregated in the cytoplasm. Furthermore, A*β* formed plaques and deposited in the extracellular matrix with the decline in A*β* clearance [36]. Alternatively, the autopsy studies in humans do not find increased amyloid and tau pathology in relation to diabetes. But many studies indicated that the A*β* aggregation was regulated by insulin signals* in vivo* and* in vitro*. Substantial evidence has suggested that insulin signaling in the brain promotes amyloidogenesis [37]. Insulin signaling directly affects A*β*PP metabolism. Once insulin has bound to its receptor, phosphoinositide 3-kinase is phosphorylated, and protein kinase B is activated, which in turn phosphorylates and thereby inhibits GSK-3. GSK-3 is a serine/threonine kinase encoded by two genes with high sequence homology (GSK-3*α* and GSK-3*β*). GSK-3*α* has an active function in A*β* production. This result implies that GSK-3 hyperactivity increases A*β* levels and contributes to AD development [38]. Also, insulin improves A*β* clearance through insulin-degrading enzyme, one of the main proteases involved in A*β* degradation [39]. In the present study, the mechanisms of A*β* deposition induced by DM remain to be elucidated in future work. In addition, our unpublished work indicated that the GSK3*β* activity increased in the hippocampus and frontal cortex of STZ-induced diabetic rats.

Diabetes is characterized by marked peripheral alterations in glucose homeostasis, which leads to hyperglycemia. Excess glucose is metabolized through alternative metabolic pathways, which may have direct adverse effects on the brain through the generation of potentially toxic metabolic by-products and through the depletion of important metabolic cofactors. In addition, diabetes is associated with long-term complications in other organ systems, wherein vascular disease can cause secondary damages to the brain. Identifying the brain injury and cognitive impairment resulting only from central nervous systemic pathology is difficult because vascular pathology and metabolic toxicity may be important in diabetic encephalopathy development. After all, more and more evidence indicates that population with the type 1 or type 2 DM are prevalence in suffering from AD. And the rodent animal model with type 1 or type 2 DM can be observed the AD pathological changes including A*β* deposition and tau-phosphorylation [40, 41].

In conclusion, aberrant metabolism including hyperglycemia and hyperlipidemia following insulin deficiency caused hippocampal atrophy, neurodegeneration, A*β* deposition, and declined dendritic spine density in STZ-induced diabetic rats. These conditions also resulted in cognitive impairment at early onset. All symptoms are characteristics of accelerated brain aging.
